# Development of Biodegradable Thermosetting Plastic Using Dialdehyde Pineapple Stem Starch

**DOI:** 10.3390/polym15183832

**Published:** 2023-09-20

**Authors:** Wasan Tessanan, Pranee Phinyocheep, Taweechai Amornsakchai

**Affiliations:** 1 Department of Chemistry, Faculty of Science, Mahidol University, Rama VI Road, Payathai, Bangkok 10400, Thailand; twasan18@gmail.com (W.T.); pranee.phi@mahidol.ac.th (P.P.); 2 Center of Sustainable Energy and Green Materials, Faculty of Science, Mahidol University, Phuttamonthon 4 Road, Salaya, Nakhon Pathom 73170, Thailand

**Keywords:** agricultural waste, pineapple stem, aldehyde starch, thermosetting plastic, biodegradable sheet, mechanical performance

## Abstract

Starch extracted from pineapple stem waste underwent an environmentally friendly modification process characterized by low-energy consumption. This process resulted in the creation of dialdehyde pineapple stem starch featuring varying aldehyde contents ranging from 10% to 90%. Leveraging these dialdehyde starches, thermosetting plastics were meticulously developed by incorporating glycerol as a plasticizer. Concurrently, unmodified pineapple stem starch was employed as a control to produce thermoplastic material under identical conditions. The objective of streamlining the processing steps was pursued by adopting a direct hot compression molding technique. This enabled the transformation of starch powders into plastic sheets without the need for water-based gelatinization. Consequently, the dialdehyde starch-based thermosetting plastics exhibited exceptional mechanical properties, boasting a modulus within the range of 1862 MPa to 2000 MPa and a strength of 15 MPa to 42 MPa. Notably, their stretchability remained relatively modest, spanning from 0.8% to 2.4%. Comparatively, these properties significantly outperformed the thermoplastic counterpart derived from unmodified starch. Tailoring the mechanical performance of the thermosetting plastics was achieved by manipulating the glycerol content, ranging from 30% to 50%. Phase morphologies of the thermoset starch unveiled a uniformly distributed microstructure without any observable starch particles. This stood in contrast to the heterogeneous structure exhibited by the thermoplastic derived from unmodified starch. X-ray diffraction patterns indicated the absence of a crystalline structure within the thermosets, likely attributed to the establishment of a crosslinked structure. The resultant network formation in the thermosets directly correlated with enhanced water resistance. Remarkably, the thermosetting starch originating from pineapple stem starch demonstrated continued biodegradability following a soil burial test, albeit at a notably slower rate when compared to its thermoplastic counterpart. These findings hold the potential to pave the way for the utilization of starch-based products, thereby replacing non-biodegradable petroleum-based materials and contributing to the creation of more enduring and sustainable commodities.

## 1. Introduction

Over the past few decades, growing environmental awareness and conscious living have brought significant attention to the global warming crisis, driven by the detrimental ecological repercussions of environmental pollution resulting from human activities. The United Nations (UN) has placed substantial emphasis on addressing these concerns by introducing the Agenda 2030 for sustainable development goals (SDGs), aiming to provide comprehensive solutions for achieving long-term environmental sustainability. This commitment has led to the emergence of three sustainable economic models collectively known as the Bio-Circular-Green (BCG) economy, which has been globally recognized and adopted across various sectors [1].

Central to the ongoing environmental challenges is the escalating accumulation of commodity plastic waste, a predicament rooted in the predominant utilization of petroleum-based or non-renewable resources for plastic production. This approach is unsustainable, primarily due to the non-biodegradable nature of most plastics. One proposed solution to alleviate this issue revolves around recycling. However, practical hurdles arise when dealing with small-sized, lightweight, and contaminated plastic waste, particularly stemming from single-use plastic products in sectors such as food delivery and personal protective equipment. These challenges often result in the escape of such waste from proper disposal cycles, leading to progressively severe environmental consequences over time [2,3].

In response to these concerns, the development of biodegradable polymer materials has gained traction, presenting a sustainable and environmentally friendly approach to address the aforementioned issues. Commercially available biodegradable plastics encompass a range of materials, including fully synthetic polybutylene adipate terephthalate (PBAT), partially biobased polybutylene succinate (PBS), fully biobased polylactic acid (PLA), as well as natural polymers like starch, polyhydroxyalkanoates (PHAs), and polyhydroxybutyrate (PHB). Each biodegradable plastic exhibits distinct characteristics, advantages, and specific biodegradability profiles within different natural environments [4,5]. For example, PBAT, PBS, and PLA find widespread applications due to their versatility, yet their decomposition in soil or marine environments is not readily achievable. These materials necessitate specialized processing facilities and disposal infrastructure, including optimal temperature, humidity, modern equipment, and specific chemical compounds, to initiate biodegradation [5,6,7]. In contrast, starch-based plastics, PHAs, and PHB offer complete biodegradability within natural settings, eliminating the need for specialized facilities. This positions them as promising alternatives to address the plastic pollution crisis, aligning with environmentally friendly and sustainable material solutions [8].

Starch, a versatile biopolymer characterized by its high abundance, low cost, renewability, and biodegradability, has garnered attention as a promising candidate for the development of bio-based plastics across various applications. A number of reviews have explored its potential in this regard [8,9,10,11,12]. Unfortunately, unmodified starch-based plastics suffer from limitations such as poor mechanical strength and low water resistance, impeding their broader utilization [13]. As a result, various strategies have been explored to mitigate these shortcomings, including chemical modifications of starch [14,15,16] and blending with other polymers [17,18,19]. Previous research has delved into various chemical reactions to modify starch, yielding oxidized starch, acetylated starch, crosslinked starch, hydroxypropyl starch, and heat-moisture treated starch [20,21,22,23,24]. These modifications impact different physicochemical properties of the resulting plastics. Among these modifications, dialdehyde starch has emerged as a noteworthy option. It serves as a crosslinking agent, obtained through controlled oxidative cleavage of the C-2 and C-3 linkages of native starch using oxidizing agents such as periodic acid and periodate, resulting in the formation of dialdehyde groups. This modified starch variant exhibits characteristics such as alkaline solubility, strong adhesion, hydrophobicity, biodegradability, and safety [25,26,27]. With these attributes in mind, our study focuses on the development of starch-based plastics utilizing dialdehyde starch.

Pineapple stem starch (PSS), a byproduct generated after bromelain extraction from pineapple stems, has attracted considerable attention due to its potential utilization in various avenues [28,29,30]. Our previous work has revealed that PSS stands out as a distinctive starch type with elevated amylose content in comparison to other variants [31]. Moreover, films derived from PSS exhibit robust mechanical strength and exceptional water resistance, while retaining biodegradability [32]. The versatility of PSS-based materials has been explored in the creation of composite sheets [33], protective coatings for fruits and vegetables [34], rigid foam [35], water-based adhesives [36], food applications [37,38], and more. This renders PSS-based plastics appealing and presents an emerging challenge for their diverse applications. Notably, no prior publications have been found on the preparation and characterization of dialdehyde pineapple stem starch (DPSS) or the utilization of aldehyde starch from PSS as a basis for starch-based plastic materials.

Consequently, our overarching goal is to elevate the range of potential applications for pineapple stem starch (PSS) through the innovation of a novel biodegradable thermosetting plastic utilizing dialdehyde pineapple stem starch (DPSS). The synthesis and detailed investigation of DPSS were undertaken with a specific focus on its physicochemical attributes. Subsequent to DPSS preparation, we proceeded to fabricate thermosetting plastic sheets, employing DPSS as the matrix and glycerol as the plasticizer. Employing a hot compression molding process devoid of water gelatinization, this approach aimed to streamline processing steps. The thorough characterization of the resultant thermoset materials encompassed evaluations of mechanical properties, morphology, crystallinity, water resistance, and biodegradability. This comprehensive understanding holds significant importance, as it may set the stage for the advancement of thermoset plastics derived from plant-based materials as a sustainable alternative to petroleum-based counterparts. By addressing environmental concerns associated with solid plastic waste, this research contributes to the pursuit of a more sustainable future.

## 2. Materials and Methods

### 2.1. Materials

The pineapple stem waste, an agricultural biomass byproduct resulting from the bromelain extraction process, was sourced from Hong Mao Biochemical Co., Ltd. located in Rayong, Thailand. Initially, the peeled pineapple stem underwent a pulverization process to disrupt the cellular structure. Subsequently, the bromelain liquid was extracted from the crushed stem through a sequence of techniques, including ultrafiltration, centrifugation, and lyophilization [39,40]. Following these steps, the residual solid material was sun-dried for several days and further ground into a fine powder using a grinder. This stem powder was then collected by sieving, utilizing an 80-mesh sieve, which effectively separated coarse fibers, cell wall components, and other solid impurities, accounting for approximately 56% of the total mass. The resulting powder was utilized without undergoing any washing process, and its characteristics closely resemble those obtained through the wet-milling procedure outlined by Nakthong et al. [31]. The composition of crude pineapple stem powder consists of three different mass fractions, including pineapple stem starch (30%), fibrous materials (56%), and other products (14%). The amylose content of pineapple stem starch is approximately (34.37 ± 2.04)% and the particle size of the starch granule is about (9.69 ± 0.02) µm. Periodic acid (99%) was procured from Shanghai Runwu Chemical Technology Co., Ltd. based in Shanghai, China. Additionally, commercial-grade ethanol and glycerol were obtained from local suppliers.

### 2.2. Preparation of Dialdehyde Pineapple Stem Starch

Dialdehyde pineapple stem starch (DPSS) was synthesized employing a modified version of the approach for dialdehyde yam starch, as outlined by Zhang et al. [41]. In this process, the initially prepared pineapple stem starch (PSS) underwent drying in a hot oven at 80 °C for 24 h prior to commencing the chemical modification procedure. Periodic acid, serving as the oxidizing agent, was weighed and introduced into a round-bottom flask. Subsequently, 500 mL of distilled water was employed to completely dissolve the periodic acid at a temperature of 40 °C. In our investigation, different concentrations of periodic acid (ranging from 0.5 mol to 1.0 mol, based on PSS) were examined to generate dialdehyde starch variants featuring varying aldehyde contents. Following this, 100 g of PSS was suspended within the periodic acid solution, and the resulting mixture underwent mechanical stirring at 40 °C for a duration of 6 h. Upon completion of the reaction, the suspended starch was filtered using filter paper and subjected to multiple washes with distilled water to eliminate any unreacted periodic acid residue. Subsequently, the modified starch underwent an additional ethanol washing step prior to being dried in a hot oven at 80 °C for 48 h until a constant weight was achieved. The final dried product was subsequently ground using a laboratory grinder and passed through a stainless steel sieve (80 mesh) to yield the desired DPSS.

### 2.3. Determination of Aldehyde Content of DPSS

The aldehyde contents in four types of DPSS prepared by varying periodic acid concentrations were determined with a rapid quantitative alkali consumption method using a titration technique [42]. The dried DPSS (0.20 g) was suspended in 10 mL of sodium hydroxide solution (0.25 mol/L) at 70 °C for 2 min and then cooled down to ambient temperature under running tap water. Subsequently, 15 mL of sulfuric acid solution (0.125 mol/L), 30 mL of distilled water, and 1 mL of 0.2% neutral phenolphthalein ethanol solution were orderly added into the DPSS solution. The mixture solution was titrated using 0.25 mol/L sodium hydroxide solution, and the end point of the titration was considered reached when the color of the solution changed from colorless to pink without further color change within 30 s. The percentage of dialdehyde unit was calculated using Equation (1) [41].

Aldehyde content (%) = ((C_1_V_1_ − 2C_2_V_2_)/((*w*/161) × 1000)) × 100 (1)

where C_1_ and C_2_ are the concentrations (mol/L) of sodium hydroxide and sulfuric acid solution, respectively. V_1_ and V_2_ are the total volume of sodium hydroxide and the sulfuric acid solution used, respectively. *w* is the dry weight of the DPSS sample. Each sample was repeated in triplicate and the sample code was defined as DxPSS, where x is the percentage of dialdehyde unit.

### 2.4. Preparation of Starch-Based Thermosetting Plastic

Thermosetting plastics were prepared from dialdehyde pineapple stem starch containing 30% aldehyde content (D30PSS) as follows: 100 g of D30PSS was added to a 500-mL beaker, and then various amounts of glycerol were weighed and added as plasticizer, according to the ratios specified in Table 1. The two components were thoroughly mixed together before being processed in a high-speed mixer (28,000 rpm) for 2 min. Each formulation of the starch mixture was then stored in a polyethylene bag at room temperature for 48 h before undergoing a hot compression molding process at 140 °C for 6 min under a pressure of 13.8 MPa to produce a 1 mm thick plastic sheet. Additionally, unmodified PSS was prepared under the same conditions to serve as a control, as indicated in Table 1.

### 2.5. Characterization of Modified Starch and Plastic

#### 2.5.1. Fourier Transform Infrared (FTIR) Spectroscopy

An FTIR spectrometer equipped with an attenuated total reflectance (ATR) accessory (Paragon 1000, PerkinElmer, Waltham, MA, USA) was used to record the ATR-FTIR spectra of the prepared dialdehyde pineapple stem starch and starch-based plastic materials. The measurement was carried out from 4000 cm^−1^ to 400 cm^−1^ with a 4 cm^−1^ resolution and 64 scans at room temperature.

#### 2.5.2. Proton Nuclear Magnetic Resonance (^1^H-NMR) Spectroscopy

The ^1^H-NMR spectra of starch samples were acquired using an Ultrashield N.M.R. spectrometer with an operating frequency of 500 MHz (Bruker Corporation, Karlsruhe, Germany). The starch samples were completely dissolved in deuterated dimethyl sulfoxide (DMSO-d_6_) as a solvent at 120 °C for 3 h and placed in NMR tubes. The chemical shifts were expressed in parts per million (ppm). The solvent peak was 2.5 ppm, and the water peak was 3.35 ppm.

#### 2.5.3. Determination of Intrinsic Viscosity

The intrinsic viscosity ([*η*]) of the starch samples with different aldehyde contents was measured using an Ubbelohde-type viscometer (capillary tube with 0.58-mm diameter) at (25 ± 0.1) °C. The starch samples were completely dissolved in dimethyl sulfoxide (DMSO) at 120 °C for 3 h to prepare the starch solution (0.05 g/dL to 0.1 g/dL). The intrinsic viscosity was determined according to the Huggins Equation (2) and Kraemer Equation (3) and each sample was performed in triplicate to obtain an average value.

*η*_sp_/C = [*η*] + *k_H_*[*η*]^2^C (2)

*ln η*_r_/C = [*η*] + *k_K_*[*η*]^2^C (3)

where C is the concentration. *k_H_* and *k_K_* are Huggins and Kraemer constants, respectively. *η*_sp_ = *η*_r_ − 1, *η*_r_ = t/t_0_; t is the efflux time of solution and t_0_ is the efflux time of solvent.

#### 2.5.4. X-ray Diffractometry (XRD)

X-ray diffraction patterns of the prepared dialdehyde pineapple stem starch and starch-based plastic materials were recorded using a benchtop X-ray diffractometer (D2 Phaser, Bruker A.X.S. GmbH, Karlsruhe, Germany) with nickel-filtered Cu Kα radiation (λ = 1.5406 Å). The scattered radiation was assessed in the angular range (2θ) of 10–40° with a step scan 15 s/point. The degree of crystallinity of the starch powder samples and starch-based plastics was calculated from the integrated peak area of amorphous and crystalline regions using Equation (4).

Crystallinity (%) = (A_c_/(A_c_ + A_a_)) × 100 (4)

where A_c_ is the integrated area of the crystalline region and A_a_ is the integrated area of the amorphous region.

#### 2.5.5. Mechanical Properties

The tensile properties of starch-based materials were investigated using a universal testing machine (Instron 5566, Instron, High Wycombe, UK) with a 1 kN static load cell capacity according to ASTM D882 [43]. Six specimens of each sample were conducted at room temperature (25 ± 2 °C) with a crosshead speed of 50 mm/min. The average values of Young’s modulus, tensile strength, and elongation at the break of starch-based plastics were received.

The hardness of the prepared plastics was evaluated using the durometer method or Shore D hardness according to ASTM D2240 [44] (GT-GS-MB model, Hardness Tester Gotech Testing Machines, Taichung City, Taiwan). The density of the materials was measured following the water displacement approach as Archimedes’ principle using a densimeter (Alfa Mirage MD-200S, Alfa Mirage, Osaka, Japan) according to ASTM D792 [45]. The average values of hardness and density were gained from five-specimen testing in each sample.

#### 2.5.6. Morphology

The tensile fractured surface of the samples was observed using a scanning electron microscope (SEM) (Hitachi SU 8000, Hitachi, Ibaraki, Japan). Before the observation, the samples were coated with platinum/palladium (Pt/Pd, 80/20).

#### 2.5.7. Water Absorption and Solubility

The water resistance of the starch-based materials was conducted in terms of the water absorption and solubility by following the procedure reported by Namphonsane et al. [35].

Water absorption: A specimen prepared from compression molding was cut to dimensions of 20 mm × 20 mm (length × width) and dried in a hot-air ventilated oven at 80 °C for 24 h. The dried weight before soaking in distilled water was recorded and defined as *w*_i_. Then, the sample specimen was immersed and gently stirred in distilled water. The absorption ability was measured at (5, 10, 20, 30, 60, 120) minutes of immersion time. Excess water on the sample surface was removed using blotting paper, and its weight was monitored and defined as *w*_f_. Water absorption was determined using Equation (5).

Water absorption (%) = ((*w*_f_ − *w*_i_)/*w*_i_) × 100 (5)

Water solubility: The measurement was assessed following the same conditions as water absorption; however, the test sample was immersed in distilled water over 24 h and after that the sample was dried at 80 °C for 24 h. The weight after drying was determined and defined as *w*_fd_. Water solubility was calculated using Equation (6).

Water solubility (%) = ((*w*_i_ − *w*_fd_)/*w*_i_) × 100 (6)

#### 2.5.8. Soil Burial Degradation Test

The biodegradability assessment of starch-based plastics was conducted using a soil burial test, following a modified protocol previously described by Seligra et al. [46]. Sample specimens with dimensions of 20 mm × 20 mm (length × width) were prepared and placed within high-density polyethylene envelopes. These envelopes were buried approximately 10 cm below the soil surface at the garden edge adjacent to the department building, with a soil pH of 7.5. The test area was shaded by trees and received regular watering. This setup provided a realistic biodegradation environment with minimal interference from the surroundings. The samples in the envelopes were visually inspected at specified time intervals to assess the level of biodegradation.

#### 2.5.9. Statistical Analysis

The statistical analysis was performed using analysis of variance (ANOVA) with the Data Analysis tool in the Microsoft Excel 2019(Office Professional Plus 2019) program. The *t*-test method with unequal variances was used to determine the statistically significant differences among the means (*p* < 0.05).

## 3. Results

### 3.1. Aldehyde Contents and Intrinsic Viscosity of DPSS

**Figure 1:**
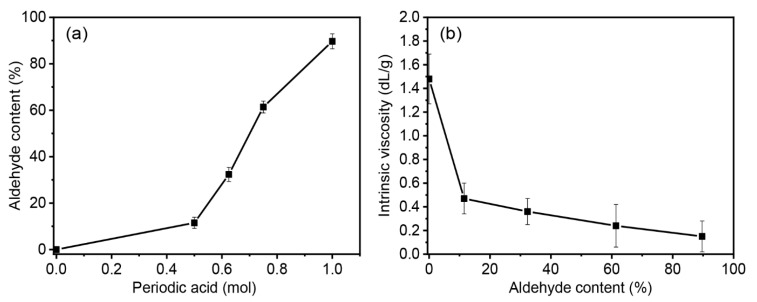
(**a**) Aldehyde contents prepared by several periodic acid concentrations and (**b**) intrinsic viscosity of the obtained DPSS at various aldehyde contents.

Pineapple stem starch was subjected to chemical modification via oxidative cleavage, employing periodic acid as the oxidizing agent, to yield dialdehyde pineapple stem starch (DPSS). The degree of oxidation, denoted by the aldehyde contents, was quantified in terms of the number of carbonyl groups per 100 glucose units. This measurement serves as an indicator of the extent of oxidation achieved under the specific conditions [47]. Notably, the aldehyde content exhibited a direct correlation with the number of carbonyl groups present, reflecting the level of oxidation imparted during the process. This relationship is visually depicted in Figure 1a and Table 2, where an increase in the periodic acid content, calculated based on the weight of PSS, resulted in a corresponding elevation of the aldehyde group concentration. The observed rise in aldehyde content is attributed to the higher oxidative degree of PSS. The achieved aldehyde contents for the respective DPSS variants were as follows: 11.50% for D10PSS, 32.25% for D30PSS, 61.37% for D60PSS, and 89.68% for D90PSS. This observation aligns with prior studies that have demonstrated an augmentation in aldehyde contents through the manipulation of the oxidizing agent content [41,47,48].

Figure 1b illustrates the intrinsic viscosity of the derived DPSS under the specific conditions and the relevant values are summarized in Table 2. In general, intrinsic viscosity serves as an indicator of starch molecular weight, with the chemical modification process inherently leading to a reduction in molecular weight [49]. As anticipated, a discernible decrease in intrinsic viscosity is evident, transitioning from 1.48 dL/g for native PSS to 0.47 dL/g for D10PSS. This trend continues as aldehyde contents increase, reaching 0.36 dL/g for D30PSS, 0.24 dL/g for D60PSS, and 0.15 dL/g for D90PSS. This phenomenon can be attributed to oxidative cleavage-induced degradation of PSS during the modification process. Periodic acid, functioning as an oxidizing agent, is adept at cleaving the C2–C3 bond positions of the anhydroglucose units within PSS. Additionally, it can break both α-D-(1–4) and α-D-(1–6) glycosidic linkages, ultimately resulting in a pronounced reduction in the intrinsic viscosity or the molecular weight of DPSS. This result is consistent with earlier findings which observed a decrease in the starch molecular weight as a consequence of oxidative cleavage modification [41,50].

### 3.2. Chemical Structure of DPSS

**Figure 2:**
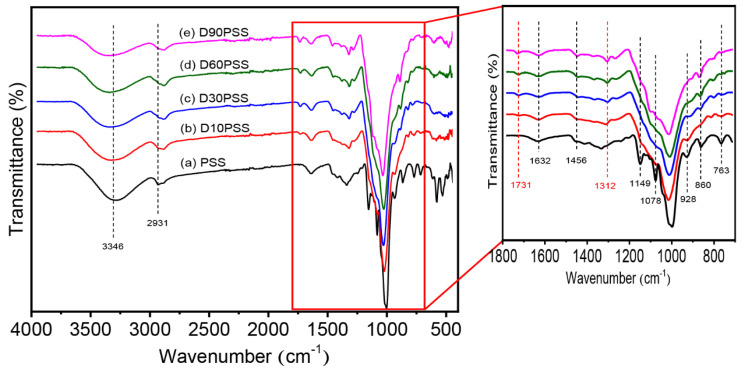
ATR-FTIR spectra of PSS and DPSS at various aldehyde contents.

The chemical structure of PSS and DPSS was investigated using ATR-FTIR analysis and the results are illustrated in Figure 2. The native PSS spectrum displayed the main vibrational peaks of O-H stretching (3346 cm^−1^), C-H stretching (2931 cm^−1^), C-O bending associated with the OH group (1632 cm^−1^), CH_2_ bending deformation (1456 cm^−1^), C-O-C stretching (1149 cm^−1^), C-O stretching (1200 cm^−1^ to 800 cm^−1^), and the C-O-C of the pyranose ring of glucose (928, 860 and 763 cm^−1^) [51,52]. The oxidative cleavage of the anhydroglucose units of native PSS using periodic acid as an oxidizing agent provided the new absorption peak at 1731 cm^−1^, associated with C=O stretching of the aldehyde functional group [53]. This follows the accepted mechanism that the C-2 and C-3 bonds in the anhydroglucose units of PSS were broken by the oxidative cleavage reaction, and the C-OH groups of starch were replaced by aldehyde groups to form modified starch containing aldehyde groups [54]. Moreover, the oxidation of anhydroglucose units was corroborated by the enhanced absorption bands at 1312 cm^−1^ and 1110 cm^−1^ [41]. The increase in aldehyde content correspondingly led to heightened intensity in the bands attributed to the aldehyde groups.

**Figure 3:**
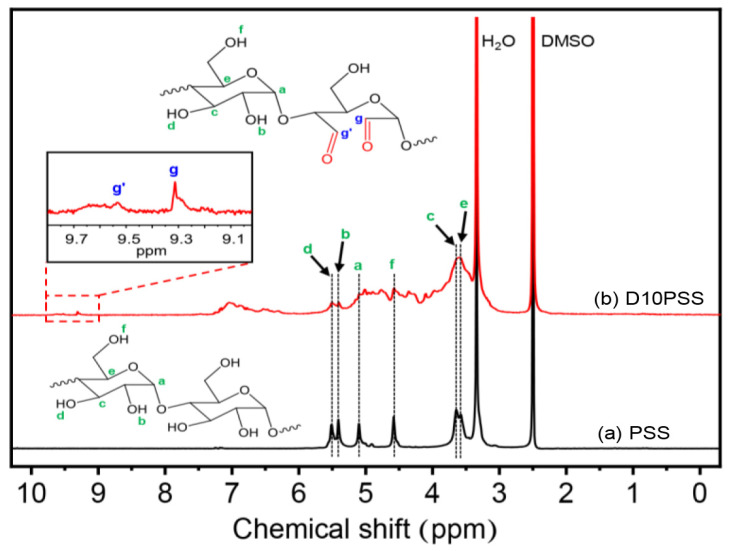
^1^H-NMR spectra of (a) PSS and (b) DPSS containing 10% of aldehyde content (D10PSS).

The chemical modification of PSS to obtain dialdehyde starch or DPSS was assured by the ^1^H-NMR technique as demonstrated in Figure 3. The native PSS displays the unique characteristic signals of the anhydroglucose unit of starch at 5.11 ppm (-**H**C_1_-O-, a), 5.41 ppm (-C_2_-O**H**, b), 3.65 ppm (-**H**C_3_-OH, c), 5.51 ppm (-CH_3_-O**H**, d), 3.58 ppm (-**H**C_5_-CH_2_-, e), and 3.6 ppm (-H_2_C-O**H**, f) [55,56]. After the oxidative cleavage modification, the dialdehyde starch displays two new signals at 9.32 ppm (g) and 9.53 ppm (g’), corresponding to the aldehyde groups of **H**C_2_=O and **H**C_3_=O, respectively [57]. Therefore, these results confirmed that DPSS was successfully prepared under the described conditions.

### 3.3. Morphological Aspects

**Figure 4:**
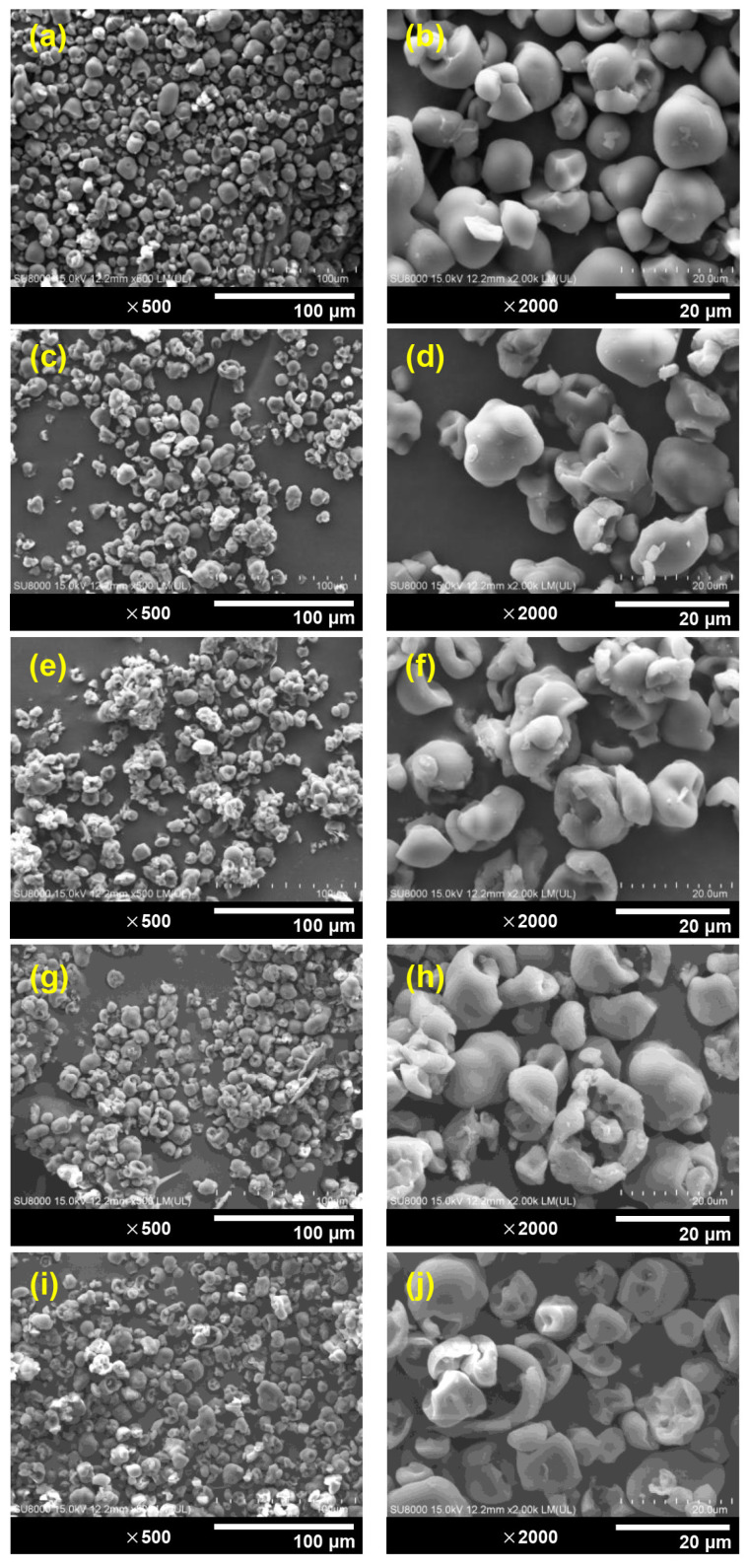
SEM images of PSS and DPSS: (**a**,**b**) native PSS, (**c**,**d**) D10PSS, (**e**,**f**) D30PSS, (**g**,**h**) D60PSS, and (**i**,**j**) D90PSS at magnifications of ×500 and ×2000.

Figure 4 presents SEM micrographs showcasing the granular morphology of both native PSS and DPSS with varying aldehyde contents. Native PSS particles (Figure 4a,b) exhibit a semi-angular shape with partially rounded features and a smooth surface, akin to findings reported by Nakthong et al. [31]. The granular structure can be attributed to the compact arrangement of PSS granules within the parenchyma cells of pineapple plant stems [58]. Following the oxidative cleavage process that yields dialdehyde starch, the appearance of modified starch particles (Figure 4c–j) distinctly diverges. All conventional DPSS particles exhibit notable morphological alterations, presenting a central circular depression. In instances where dialdehyde starch carries higher carbonyl contents, the distortion becomes notably pronounced, accompanied by a larger circular depression. This phenomenon aligns with prior observations regarding dialdehyde materials derived from diverse starch types [41,53,59,60]. The underlying cause could be linked to the localization of amorphous regions predominantly within the internal area of starch particles, with crystalline regions distributed around the exterior. The non-crystalline segments of PSS particles are more susceptible to oxidation by periodic acid, particularly when higher quantities of this oxidizing agent are employed to achieve increased oxidation levels. This leads to greater internal corrosion of the dialdehyde starch [61]. The resulting DPSS particles also exhibit a densely conglomerated appearance, with granules growing in size in contrast to the native PSS. This phenomenon points to significant depolymerization and surface oxidation, fostering heightened interactions between starch particles [62].

**Figure 5:**
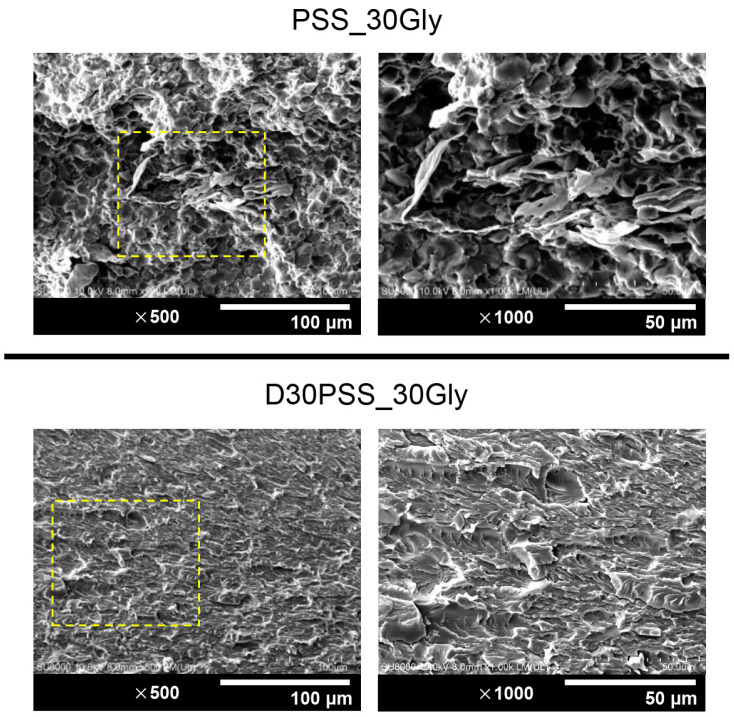
SEM images of tensile fractured surface of starch-based plastic materials containing 30 wt.% glycerol prepared from PSS and D30PSS at magnifications of ×500 and ×1000. The yellow boxes indicate the corresponding area of the ×1000 images.

The thermosetting plastic starch was produced using D30PSS with glycerol as the plasticizer. Simultaneously, the thermoplastic plastic starch derived from pristine PSS was prepared under identical conditions to serve as a comparative sample against the modified starch. Figure 5 displays SEM images capturing the fractured surfaces of all starch-based plastics obtained after the tensile test. Regions of interest within starch-based plastic microstructures are denoted by yellow-dotted rectangular boxes, shown at a higher magnification (×1000).

Consequently, the thermoplastic starch derived from PSS containing 30% of the weight fraction of glycerol (PSS_30Gly) exhibited a heterogeneous microstructural phase, distinguishing between starch granules and the glycerol-plasticized starch phase. In contrast, the thermosetting plastic starch derived from D30PSS containing 30% of the weight fraction of glycerol (D30PSS_30Gly) revealed a homogeneous structure, featuring a rough surface devoid of residual starch particles. This phenomenon can be elucidated through two underlying mechanisms: Firstly, glycerol, functioning as a plasticizer, typically disrupts inter- and intramolecular hydrogen bonds within starch granules to facilitate plasticization [63]. Secondly, aldehyde starch can participate in the formation of inter- and intramolecular acetal and hemiacetal linkages during the preparation of starch-based plastics, influenced by elevated temperature and glycerol content [25].

### 3.4. X-ray Diffraction (XRD)

**Figure 6:**
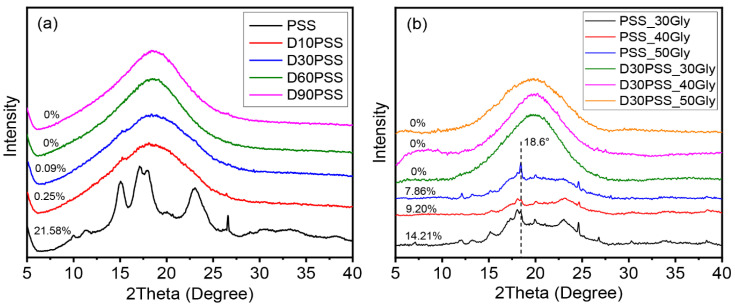
(**a**) XRD diffraction pattern of granular starch samples and (**b**) starch-based plastic materials. Schemes follow the same formatting.

XRD diffraction patterns of native PSS and DPSS variants with differing aldehyde contents, along with starch-based plastics plasticized with varying glycerol quantities, are presented in Figure 6. Starch granule crystal polymorphs are typically categorized into three types based on XRD patterns: A-type, B-type, and C-type crystalline structures [64]. Illustrated in Figure 6a, native PSS exhibited robust peaks approximately at 2θ values of (15.0°, 17.1°, 18.0°, 23.0°), along with fainter peaks around 2θ values of (10.0°, 11.3°, 20.1°, 26.6°, 30.2°, 34.0°), indicative of the A-type crystalline arrangement. This outcome mirrors findings concerning various starch types as reported by Nakthong et al. [31] and the corn starch documented in the PDF catalog (Reference #39-1911) [65]. The native PSS displayed a crystallinity of approximately 21.58%. Following the oxidative cleavage of PSS, DPSS showcased a marked reduction in crystalline peaks (D10PSS and D30PSS), transforming into a broadened peak at around 18.49°, devoid of specific crystal patterns (D60PSS and D90PSS). The degree of crystallinity for DPSS is detailed in Figure 6a. This outcome can be attributed to the disruption of PSS’s crystalline structure during oxidative cleavage modification with periodic acid under acidic conditions [66].

Turning attention to the crystalline structure of starch-based plastic sheets derived from PSS and D30PSS, with varying glycerol contents, it is depicted in Figure 6b. XRD patterns of PSS-based plastics, produced using the hot compression molding technique, retained similarities to native PSS powder’s A-type polymorph [31]. Notably, a discernible decline in diffraction intensity ensued post-glycerol plasticization. This divergence stands in contrast to PSS films created through solution casting methods [34,35]. Additionally, an emerging peak around 18.6° is observed, likely linked to the V-type crystalline structure. This emergence may be attributed to the formation of amylose-glycerol complexes during starch plasticization under elevated temperature and pressure [67]. The degree of crystallinity experienced a decline with augmented glycerol content [33]. Analyzing thermosetting plastic starch from D30PSS, XRD diffractograms showcased broader and weaker amorphous peaks. This phenomenon indicates that D30PSS-based plastics can foster inter- and intramolecular crosslinking under elevated temperature and pressure, thereby adopting the characteristics of typical amorphous or thermosetting plastics without recrystallization [53].

### 3.5. Mechanical Properties

**Figure 7:**
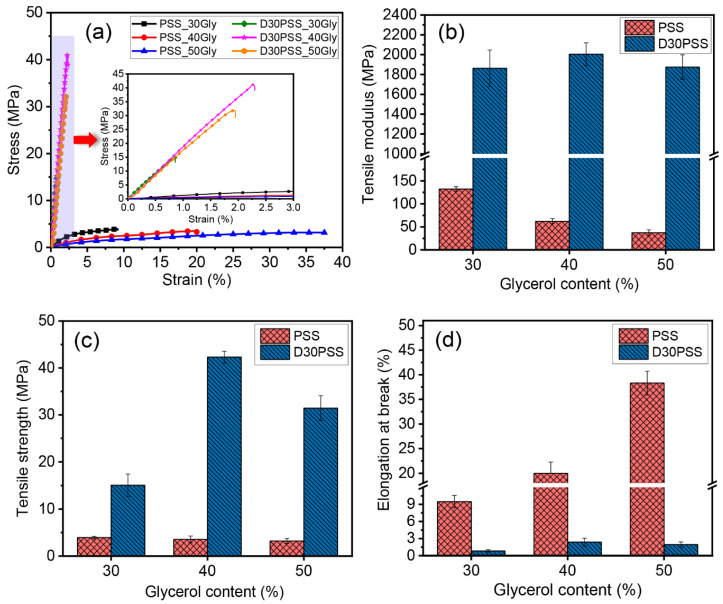
Tensile properties of starch-based plastic materials prepared by PSS and D30PSS containing different amounts of glycerol: (**a**) stress-strain curves, (**b**) tensile modulus, (**c**) tensile strength and (**d**) Elongation at break.

Figure 7 depicts the tensile properties of starch-based plastics derived from PSS and D30PSS with varying glycerol contents, and the relevant values are listed in Table 3. As shown in Figure 7a, stress–strain curves of D30PSS-based thermosetting plastics, with different glycerol concentrations, reveal intriguingly higher stress values compared to PSS-based thermoplastics. However, this enhancement in stress is accompanied by a significant reduction in stretchability. In the case of PSS-based plastics, greater glycerol content led to a gradual decrease in stress but an increase in strain at break due to glycerol’s plasticizing effect. Conversely, thermosetting plastic starch or D30PSS-based plastics exhibited a marked increase in stress and a substantial decrease in extensibility as higher glycerol content was introduced. Figure 7b–d illustrate the average trends in tensile performance.

**Figure 8:**
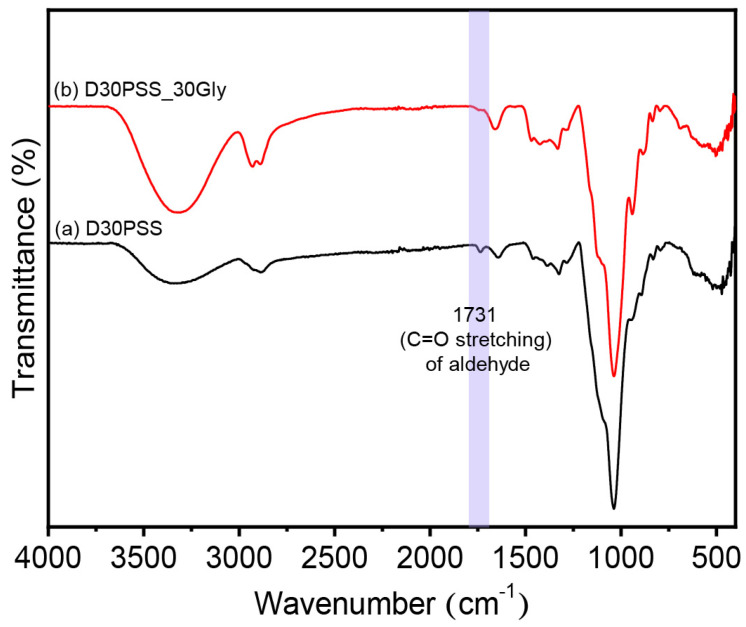
ATR-FTIR spectra of (a) the starting D30PSS powder and (b) D30PSS_30Gly thermosetting plastics.

**Figure 9:**
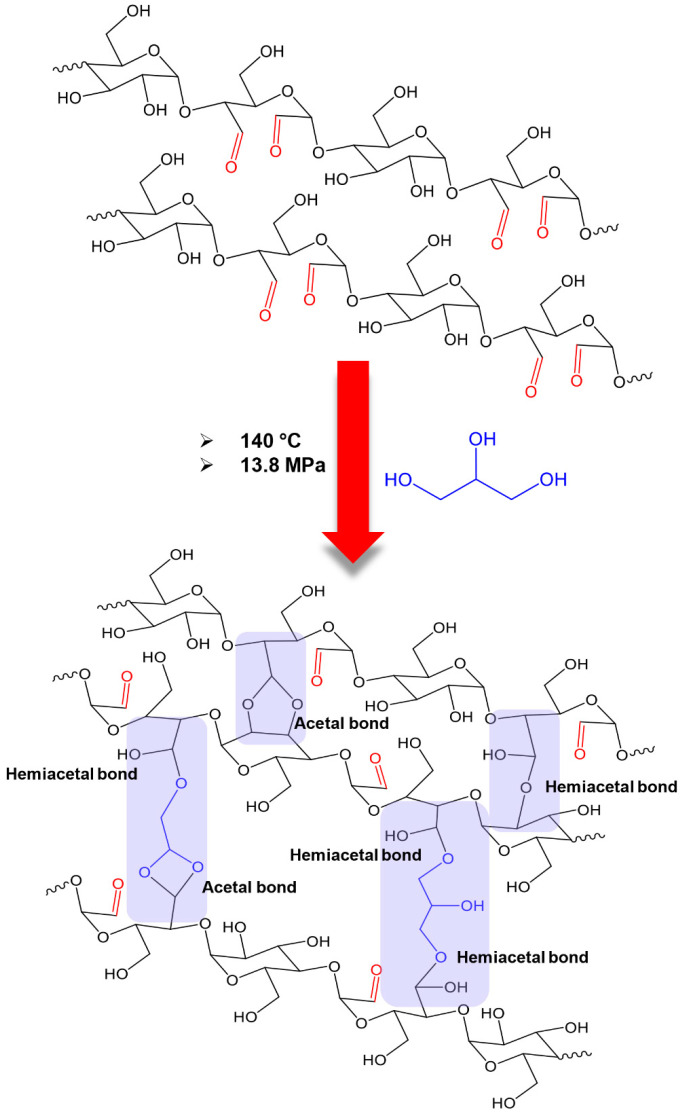
The proposed linkage structure of thermosetting plastic prepared from dialdehyde pineapple starch under the hot compression molding process.

The tensile modulus of thermosetting plastic produced via hot compression molding ranged from approximately 1862 MPa to 2000 MPa, with tensile strength around 15 MPa to 42 MPa, and strain at break ranging from 0.8% to 2.4%. In contrast, PSS-based thermoplastics, prepared using the same method, displayed a modulus of about 37 MPa to 132 MPa, tensile strength around 3.2 MPa to 3.9 MPa, and elongation capability spanning 9% to 38%. This suggests that the thermosetting plastic generated could form an inter- and intramolecular acetal and hemiacetal crosslinking network during the hot compression molding process for sheet production [25,26]. Furthermore, as previously reported, aldehyde materials can establish network linkages with hydroxyl functional groups [68,69,70]. Thus, while glycerol acts as a plasticizer, it may also engage in acetal linkage formation with dialdehyde starch. To validate this, Figure 8 presents the ATR-FTIR spectrum of D30PSS_30Gly plastic compared to D30PSS powder. A noticeable reduction in C=O aldehyde absorption intensity at 1731 cm^−1^ confirms the utilization of aldehyde groups in the modified starch to form crosslinking linkages during thermosetting plastic formation. The proposed linkage structure of thermosetting plastic derived from dialdehyde pineapple starch is visualized in Figure 9. Notably, the highest strength values achieved by the thermosetting plastic, prepared using dialdehyde pineapple starch in this study, surpass those of other aldehyde starch-based plastics, which typically fall within the range of 2 MPa to 30 MPa [53,69].

**Figure 10:**
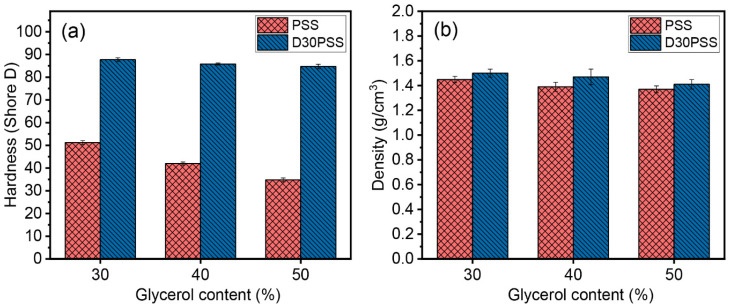
(**a**) Hardness (Shore D) and (**b**) density of starch-based plastic materials prepared by PSS and D30PSS containing different amounts of glycerol.

Figure 10a displays the hardness (Shore D) of starch-based materials containing different amounts of glycerol. Thermosetting plastics, specifically D30PSS-based plastics, exhibit hardness values ranging from approximately 85 to 88 Shore D, while PSS-based plastics record hardness values around 35 to 51 Shore D. Notably, hardness values show a decreasing trend with higher glycerol contents. As anticipated, the prepared thermosetting plastics exhibit greater hardness compared to conventional thermoplastic materials.

The densities of the starch-based plastics were also monitored, as depicted in Figure 10b. The trends in values mirrored those of the hardness results. Thermosetting starch materials (1.41 g/cm^3^ to 1.50 g/cm^3^) exhibited higher density values than their thermoplastic counterparts (1.37 g/cm^3^ to 1.45 g/cm^3^). Additionally, increasing the amount of glycerol content led to a decrease in the materials’ density. This trend aligns with expectations, as the strong molecular packing facilitated by the covalent network in thermosetting plastics results in higher density compared to thermoplastic materials [71].

### 3.6. Water Absorption and Solubility

**Figure 11:**
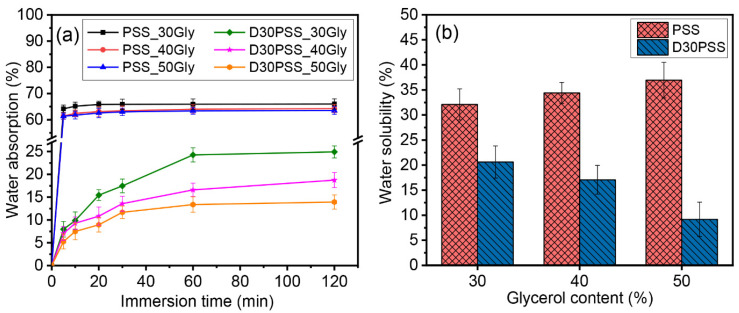
(**a**) Water absorption and (**b**) water solubility of starch-based plastic materials prepared by PSS and D30PSS containing different amounts of glycerol.

Figure 11 demonstrates the water absorption and solubility of starch-based plastic materials prepared from PSS and D30PSS with varying amounts of glycerol. As depicted in Figure 11a, the water absorption capacity of all samples increases with soaking time, eventually plateauing after a certain period. The stable points of water-absorbing ability are influenced by the presence of glycerol. Gradually increasing glycerol content leads to a decline in the maximum water absorption capacity, similar to observations previously reported for PSS film [33]. Other studies also note a decrease in water absorption capacity with higher plasticizer concentrations in carbohydrate-based plastics [72,73,74]. This can be explained by the strong hydrogen bonding between starch and glycerol, which hinders the interaction of water molecules with starch or plasticizer. Higher glycerol concentrations foster more robust hydrogen bonding between starch and plasticizer [75].

The water solubility of all starch-based plastics is depicted in Figure 11b. Ensuring water insolubility is crucial for extending the shelf life of plastic products [76]. The water solubility of D30PSS-based plastics gradually decreases, while that of PSS-based plastics increases with higher glycerol content. Consequently, the crosslinked plastic prepared from D30PSS exhibits enhanced water stability with reduced water-soluble characteristics (9% to 21%). This effect is attributed to the presence of crosslinking chains in the plastic, significantly improving water insolubility [21,68]. Similarly, in the case of thermosetting starch plastic, higher glycerol content enhances water insolubility, consistent with the proposed crosslinking structure as shown in Figure 9. Conversely, PSS-based plastics show a gradual increase in solubility (32% to 37%) with rising glycerol concentration, a trend noted in previous reports [33,75,77]. This behavior aligns with the findings of Basiak et al. [78], where starch with higher amylose content exhibited a higher water solubility index.

### 3.7. Soil Burial Degradability

**Figure 12:**
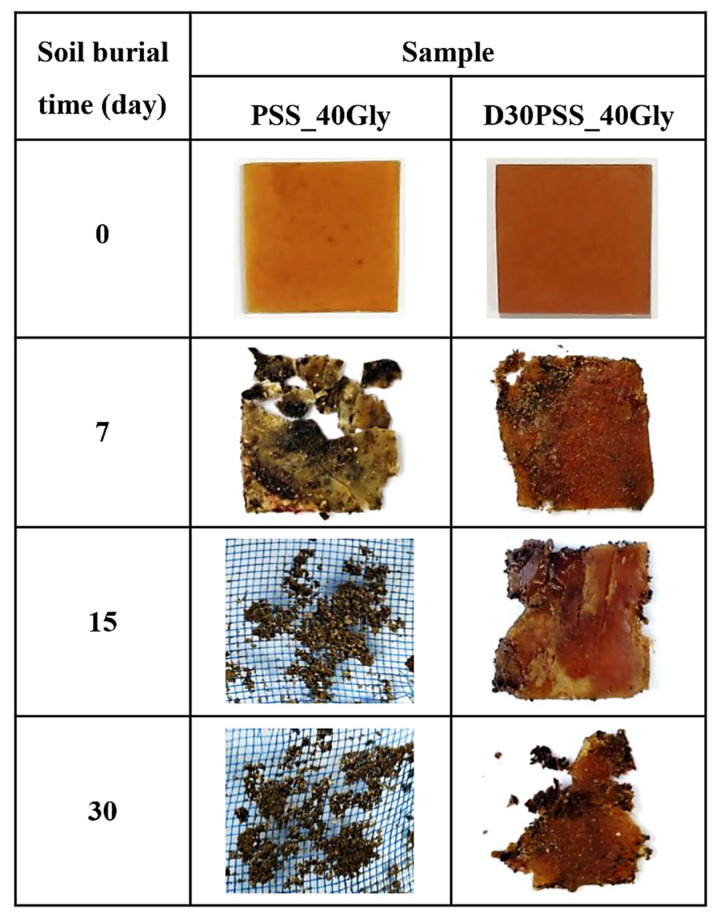
Photographs of some starch-based plastic materials prepared by PSS and D30PSS before and after soil burial degradation with different periods of time.

Figure 12 showcases photographs of select starch-based plastics prepared from PSS and D30PSS with 40% glycerol content before and after undergoing a soil burial degradation test. Throughout the test, all plastic products underwent evident deterioration, albeit at varying rates over time. Notably, the observations were as follows: after 7 days, the PSS_40Gly plastic exhibited signs of mold growth, fragmented into smaller pieces, and completely decomposed by the 15th day. On the other hand, the D30PSS_40Gly plastic sample sheet appeared slightly swollen and showed minor degradation after 7 days, while retaining its original shape. Subsequently, it disintegrated into smaller fragments after 15 and 30 days. As anticipated, the crosslinked starch systems demonstrated prolonged degradation times [79].

## 4. Discussion

The preparation of dialdehyde pineapple stem starch with varying aldehyde contents was successfully demonstrated by adjusting periodic acid concentrations, as confirmed by the chemical structure through ATR-FTIR and ^1^H-NMR analyses. The aldehyde groups were found to be randomly distributed along the anhydroglucose backbone of starch. Moreover, the oxidative cleavage modification under acidic conditions led to the breakdown of starch molecular chains, reducing the modified starch’s molecular weight as visualized in a significant decrement in the intrinsic viscosity value by adding an oxidizing agent and a more drastic decrease by increasing the periodic acid concentration (0.5 mol to 1 mol). This result is consistent with previous research, which described an apparent decline in starch molecular weight undergoing an oxidative cleavage modification [41,50].

By combining the prepared dialdehyde starch with glycerol, the mixture was subjected to high temperature and pressure to create a three-dimensional network material known as thermosetting plastic. The formation of a crosslinked network in the thermosetting starch products was evident from multiple indications. The FTIR signal of the aldehyde group diminished, indicating the formation of linkages. Additionally, there was an increase in hardness and mechanical properties, suggesting the establishment of a stronger network structure. Furthermore, the reduced water absorption and solubility further supported the presence of intermolecular linkages within the material.

Notably, the starch-based thermoset plastic prepared from dialdehyde pineapple starch exhibited an impressive mechanical performance with the maximum value of modulus (2004.08 ± 114.82 MPa) and mechanical strength (42.31 ± 1.26 MPa). This can be compared with previous findings on a similar topic reported by Yu et al. [53], who prepared dialdehyde starch from potato starch and used it for fabricating starch-based plastics. They revealed the mechanical strength of a plastic product of only approximately 2.5 MPa using dialdehyde starch containing 30% aldehyde content and 40% (by weight) of glycerol as a plasticizer. Moreover, Zhang et al. [69] also demonstrated the mechanical properties of the dialdehyde starch-based plastics. They found that combining dialdehyde starch (40% aldehyde content) and 30% of weight fraction of glycerol to fabricate starch-based plastic suggested a mechanical strength of only about 2.4 MPa. Thermosetting plastic (9% to 21% water solubility) showed a higher water resistance than thermoplastic starch obtained from unmodified pineapple stem starch (32% to 37% water solubility). This research revealed the higher efficacy of thermosetting pineapple stem starch materials for water-resistant ability compared to pineapple stem starch-based plastic reported by Namphonsane et al. [32] and Thongphang et al. [33], which revealed a water resistance of (17 to 25)% and (15 to 32)%, respectively, depending on glycerol content. Furthermore, the thermosetting starch displayed a slowed biodegradation rate while still retaining biodegradability. These findings collectively highlight the successful transformation of pineapple stem waste into valuable thermosetting plastic materials with enhanced mechanical properties and reduced environmental impact.

As a result, these prepared thermosetting materials hold promise as viable options for single-use or disposable products, as well as other plastic applications such as film coatings, trays, and containers. While this study has shed light on the network structure, further comprehensive investigations will be required and are planned for future publications.

## 5. Conclusions

The dialdehyde pineapple stem starch was successfully synthesized using a green approach involving periodic acid as an oxidizing agent, characterized by eco-friendly conditions and minimal energy consumption. The prepared dialdehyde pineapple stem starch with several aldehyde contents (10, 30, 60, 90)% was acquired by varying periodic acid concentrations (0.5 mol to 1.0 mol). Through the method of hot compression molding, thermosetting plastic sheets were successfully manufactured from this modified starch and glycerol, offering a cost-effective and practical fabrication technique. Notably, the mechanical attributes of the thermosetting materials show a higher value of modulus (~2004 MPa) and mechanical strength (~42 MPa) than those of thermoplastic starch prepared from the unmodified pineapple starch, rendering them suitable for a wide array of end-use applications. Furthermore, the thermosetting products derived from dialdehyde pineapple stem starch demonstrated exceptional water resistance and biodegradability, positioning them as viable contenders for diverse packaging solutions. This newfound knowledge holds the potential to foster a circular economy and mitigate carbon emissions, leveraging pineapple stem waste as a substantial renewable resource to yield valuable materials. By combining innovation with sustainability, this research contributes to the broader objective of addressing environmental challenges and promoting the transition towards a more responsible and resource-efficient future.

## Figures and Tables

**Table 1:** Sample formulations of the pineapple stem starch-based plastic material.

Sample Code	Starch (Weight Fraction, %)		Glycerol (Weight Fraction, %)
	PSS	D30PSS	
PSS_30Gly	100	-	30
PSS_40Gly	100	-	40
PSS_50Gly	100	-	50
D30PSS_30Gly	-	100	30
D30PSS_40Gly	-	100	40
D30PSS_50Gly	-	100	50

**Table 2:** Aldehyde content and intrinsic viscosity of the obtained DPSS prepared in this work.

Sample Code	Aldehyde Content (%)	Intrinsic Viscosity (dL/g)
PSS	-	1.48 ± 0.21 ^a^
D10PSS	11.5 ± 2.39 ^d^	0.47 ± 0.13 ^b^
D30PSS	32.35 ± 3.06 ^c^	0.36 ± 0.10 ^b^
D60PSS	61.37 ± 2.55 ^b^	0.24 ± 0.18 ^b^
D90PSS	89.68 ± 3.22 ^a^	0.15 ± 0.13 ^b^

Mean ± SD values with different letter superscripts within in the same column refer statistically significant differences among the means (*p* < 0.05).

**Table 3:** Mechanical properties of the pineapple stem starch-based plastic materials.

Sample Code	Tensile Properties			Hardness (Shore D)	Density (g/cm^3^)
	*E* * (MPa)	σ ** (MPa)	ε *** (%)		
PSS_30Gly	132.42 ± 5.06 ^b^	3.92 ± 0.26 ^d^	9.48 ± 1.07 ^c^	51.20 ± 0.84 ^d^	1.45 ± 0.03 ^a^
PSS_40Gly	62.19 ± 5.68 ^c^	3.52 ± 0.74 ^d^	20.00 ± 2.27 ^b^	42.00 ± 0.71 ^e^	1.39 ± 0.04 ^b^
PSS_50Gly	37.24 ± 6.35 ^d^	3.19 ± 0.53 ^d^	38.33 ± 2.41 ^a^	34.80 ± 0.83 ^f^	1.37 ± 0.03 ^c^
D30PSS_30Gly	1862.03 ± 184.35 ^a^	15.08 ± 2.38 ^c^	0.83 ± 0.21 ^e^	87.70 ± 0.84 ^a^	1.50 ± 0.03 ^a^
D30PSS_40Gly	2004.08 ± 114.82 ^a^	42.31 ± 1.26 ^a^	2.38 ± 0.69 ^d^	85.80 ± 0.45 ^b^	1.47 ± 0.06 ^a^
D30PSS_50Gly	1874.93 ± 124.24 ^a^	31.45 ± 2.63 ^b^	1.95 ± 0.46 ^d^	84.70 ± 0.97 ^c^	1.41 ± 0.04 ^b^

Mean ± SD values with different letter superscripts within in the same column refer to statistically significant differences among the means (*p* < 0.05). * Tensile modulus, ** Tensile strength, *** Elongation at break.

## Data Availability

The data presented in this study are available on request from the corresponding author.
